# Supplementary material for: Social support, distress, stress, anxiety, and depression as predictors of suicidal thoughts among selected university students in Bangladesh
Source: PLOS Glob Public Health. 2024 Apr 16;4(4):e0002924. doi: 10.1371/journal.pgph.0002924 (PMC11020522; doi:10.1371/journal.pgph.0002924)
Supplement: Attachment — Submitted filename: Reviewer report suicidal thought.docx [file pgph.0002924.s002.docx]

Dear Editor-in- Chief,

I have provided my comments on the manuscript, **PGPH-D-23-02566** entitled,” **Association of social support with suicidal thoughts among a young adult university student sample in Bangladesh: The mediating role of distress, stress, anxiety, and depression”**

The manuscript examined the association of perceived social support with suicidal thoughts among a young adult sample of university students and estimates the degree to which perceived stress, distress, anxiety, and depression may explain the association using survey method. The manuscript is generally well written and clearly presented.

Other general and specific comments are provided below:

1. Originality of value: The manuscript presents an original study which was designed and conducted among young adult university students in Bangladesh. The paper addressed a relevant public health issue of concern, namely suicidal thought and with thought-provoking results.

2. Suitability and soundness of technique: The techniques used for the study are adequate and robust. It utilized a cross-sectional research design and questionnaire.

3. Clarity of Presentation: The manuscript was exceptionally clear in narrative presentation. It is easy to follow and complied with the journal specifications.

4. Areas requiring correction. While the paper is generally well written, some corrections are required, which have been highlighted for the authors' attention below

**Recommendation:** The paper should be accepted with minor correction

Thank you.

Yours sincerely,

**Olaoye A. Titilayo PhD**

**Abstract**

The authors should include the information on the instrument, validation, and sampling technique in the abstract.

**Methods**:

The authors should describe in detail and clearly how they selected the participants from the faculties and the number of participants per departments and the sampling frame.
