# Supplementary material for: Social support, distress, stress, anxiety, and depression as predictors of suicidal thoughts among selected university students in Bangladesh
Source: PLOS Glob Public Health. 2024 Apr 16;4(4):e0002924. doi: 10.1371/journal.pgph.0002924 (PMC11020522; doi:10.1371/journal.pgph.0002924)
Supplement: Attachment — Submitted filename: Response to the Reviewer(s).docx [file pgph.0002924.s003.docx]

February 25, 2024

Nnodimele Onuigbo Atulomah, PhD
Academic Editor
PLOS Global Public Health

**Re: Manuscript Number** PGPH-D-23-02566

Dear Dr. Nnodimele Onuigbo Atulomah,

Thank you for considering and giving us the chance to submit the revision of our manuscript, **Social support, distress, stress, anxiety, and depression as predictors of suicidal thoughts among selected university students in Bangladesh,** for possible publication in PLOS Global Public Health. We have revised the manuscript according to the comments by the editor and the reviewers. Their useful and productive comments helped us improve the clarity and quality of the manuscript. Where we have changed the text, the corresponding sentences in the text have been highlighted in the track changes. Where textual changes are included in this response to the reviewer, we have indented and italicized them and removed references.

We hope that the revisions are satisfactory in addressing issues raised by the reviewers and look forward to hearing your decision about this article.

Yours sincerely,

Corresponding Author

Journal Requirements:

1. Please review your reference list to ensure that it is complete and correct. If you have cited papers that have been retracted, please include the rationale for doing so in the manuscript text, or remove these references and replace them with relevant current references. Any changes to the reference list should be mentioned in the rebuttal letter that accompanies your revised manuscript. If you need to cite a retracted article, indicate the article’s retracted status in the References list and also include a citation and full reference for the retraction notice.

**Response:** Thank you very much. We have corrected and updated the references.

2. Please provide separate figure files in .tif or .eps format only and remove any figures embedded in your manuscript file. Please also ensure all files are under our size limit of 10MB.

**Response:** Thank you very much. We have updated the figure in .tif format and uploaded the updated version.

Additional Editor Comments (if provided):

The two independent reviewers appointed to evaluate this manuscript both agreed that the study represents valuable contribution to the field and made recommendations for revisions. However I think that the title is too long and should be modified to "Social-support, distress, stress, anxiety, and depression as predictors of suicidal thoughts among selected university students in Bangladesh". Kindly attend to the recommendations made by the reviewers.

**Response:** Thank you. We have changed the title according to your suggestion in the revised version.

Reviewers' comments:

**Reviewer #1:** The authors have scientifically examined the association of perceived social support with suicidal thoughts among a young adult sample of university students, estimated the degree to which perceived stress, distress, anxiety, and depression may explain the association, and concluded that perceived social support reduces the likelihood of suicidal thoughts among university students and that mental disorders have a partial mediating effect on the association.
The authors were able to determine not only the main association (social support predicting suicidal thoughts) but also the contribution of each potential mediator(stress, distress, anxiety, and depression), both individually and collectively, to suicidal thoughts. This is highly commendable.

**Response:** Thank you very much.

The study is timely and globally relevant; when published, it will provide evidence to institutions of higher learning, families, clinicians, and health regulatory agencies to adopt strategies that will intentionally provide social support to students with adverse mental health outcomes and reduce incidences of suicidal thoughts, suicidal attempts, and suicide. The recommendations will also enhance the mental well-being of university students if implemented.

**Response:** Thank you very much for your inspirational comments.

The authors are to address the following:

• There was no indication of the type of survey instrument used (online or paper survey instruments, the authors only reported that 642 randomly selected students were interviewed after giving their consent, but did not indicate how the interview was undertaken.

**Response:** Thank you. It was paper survey. We have re-write the first line of method section as follows for more clarity.

*“**A cross-sectional study was conducted from June to September 2022, utilizing a paper-based survey among students of the University of Rajshahi, Bangladesh.”*

• The sample size was increased to 642 to strengthen the survey. Noting that the type of survey instrument was not indicated, it was not clear to the reviewer if the 642 participants recruited all gave their responses, and that the response rate was 100%. It is necessary to clarify this because the same number of selected students (642) were included in the analysis.

**Response:** Thank you for your insightful comment. We forgot to provide this information. It was unintentional mistake. We apologize. We have added the following sentence for better describing the chosen sample and making it clear.

*“However, a total of 700 students were invited to participate in this study during the study period; of these, 32 were absent, 18 were unable to attend due to schedule constraints, and 8 did not complete all the items of the questionnaire. This resulted in a final sample of 642 participants, yielding a response rate of 92%.”*

• While the discussion section is expected to analyze the findings of the study and relate them with those of previously published similar studies, authors should avoid repeating results in the discussion section.

**Response:** Thank you very much. We have just highlighted the summary findings in the first paragraph of the discussion section for better understanding by the readers. In the 2^nd^ and 3^rd^ paragraphs, we compared our findings with other similar research findings and also provided possible explanations of our findings. So, we think the discussion section is written according to your suggestion.

**Reviewer #2:** The manuscript examined the association of perceived social support with suicidal thoughts among a young adult sample of university students and estimates the degree to which perceived stress, distress, anxiety, and depression may explain the association using survey method. The manuscript is generally well written and clearly presented.
Other general and specific comments are provided below:

1. Originality of value: The manuscript presents an original study which was designed and conducted among young adult university students in Bangladesh. The paper addressed a relevant public health issue of concern, namely suicidal thought and with thought-provoking results.
2. Suitability and soundness of technique: The techniques used for the study are adequate and robust. It utilized a cross-sectional research design and questionnaire.
3. Clarity of Presentation: The manuscript was exceptionally clear in narrative presentation. It is easy to follow and complied with the journal specifications.
4. Areas requiring correction. While the paper is generally well written, some corrections are required, which have been highlighted for the authors' attention below
Recommendation: The paper should be accepted with minor correction

**Response:** Thank you very much for your good, positive, and valuable comments. We accommodated in our revised manuscript what you asked us to include in the abstract and method sections.

Abstract
The authors should include the information on the instrument, validation, and sampling technique in the abstract.

**Response:** Thank you. We have revised it according to your suggestion.

Methods:
The authors should describe in detail and clearly how they selected the participants from the faculties and the number of participants per departments

**Response:** Thank you very much. The following sentences were added in the revised manuscript.

*“Two-stage sampling procedures were used to collect the data. There are 12 faculties at the University of Rajshahi, with varying numbers of students. At the first stage, samples were allocated to each faculty, with the probability proportional to the size of the students. At the second stage, one department from each of the 12 faculties was chosen at random, and the assigned samples for each faculty were then distributed to each of the selected departments. All students from the selected departments were eligible for this study.”*
